# A Medical Officer's Libel Action

**Published:** 1917-12-15

**Authors:** C. A. W. Roberts

**Affiliations:** Master. West Derby Union. Institution: Walton-On-the-Hill, Liverpool


*To the Editor of* The Hospital.

### A Medical Officer's Libel Action.

*To the Editor of* The Hospital.

Sir,—As a regular reader of your journal The Hospital, I should like to point out that, in your comments on a recent libel case at Steppinghill Hospital, you make the statement that Dr. Collingwood Fenwick is the first resident medical officer, and also that the hospital is still attached to the workhouse.

I should like you to correct this, as I was appointed steward there in 1905, and worked in the most amicable co-operation with no less than three resident medical officers, under the supervision of the late Dr. W. Barker Baile (who died some twelve months ago), and the same matron who at present holds the office.

The administration of the hospital is entirely separate from that of the workhouse, and, although it is seven or eight years since I left there, I have the most pleasant memories of my association with the medical men and the matron who worked in that institution.—Yours faithfully,

C. A. W. Roberts, Master.

West Derby Union.
Institution: Walton-on-the-Hill,
Liverpool, December 7, 1917.

[Mr. Roberts' letter calls attention to some among the confusions which were apparent to anyone who read the reports of the case. In summarising it we felt bound, however, to follow the statements of counsel as reported in the local newspapers. For instance, Mr. Langdon, in his opening speech for the plaintiff, is reported to have said, "Previously"—that is before Dr. Fenwick's appointment—"the hospital had been run as a Poor-Law hospital without a resident medical officer." Secondly, so long as the proposals of a medical officer have to be sub-

**EDITOR'S LETTER BOX**—(*continued*).

mitted to a Board of Guardians an infirmary is not wholly under medical control. In fact, at present one of the most important qualifications of a Poor-Law infirmary superintendent is a gift for managing Boards of Guardians. But, as Mr. Roberts points out, notwithstanding the difficulties, pleasant associations are happily frequent.—Ed. The Hospital.]

